# Diverse cardiac phenotypes among different carriers of the same *MYH7* splicing variant allele (c.732+1G>A) from a family

**DOI:** 10.1186/s12920-022-01186-z

**Published:** 2022-02-24

**Authors:** Peng Tu, Hairui Sun, Xiaohang Zhang, Qian Ran, Yihua He, Suzhen Ran

**Affiliations:** 1 Department of Ultrasound, Chongqing Health Center for Women and Children, 120 Longshan Road, Yubei District, Chongqing, 401147 China; 2 Maternal-Fetal Consultation Center of Congenital Heart Disease, Department of Echocardiography, Beijing Anzhen Hospital, Capital Medical University, 2 Anzhen Road, Chaoyang District, Beijing, 100029 China

**Keywords:** Left ventricular non-compaction, *MYH7*, Splice site mutation

## Abstract

**Background:**

Left ventricular non-compaction cardiomyopathy (LVNC) is a rare congenital heart defect. Gene defections have been found in patients with LVNC and their family members; and *MYH7* is the most frequent gene associated with LVNC.

**Methods:**

We performed a complete prenatal ultrasound and echocardiographic examination on a fetus with cardiac abnormality and a parent–child trio whole-exome sequencing to identify the potential genetic causes. When the genetic abnormality in MYH7 was identified in the fetus, we performed echocardiography and genetic screening on its high-risk relatives.

**Results:**

Second trimester ultrasound and echocardiography showed several malformations in the fetus: Ebstein’s anomaly (EA), heart dilatation, perimembranous ventricle septal defects, mild seroperitoneum, and single umbilical artery. Heterozygous genotyping of a splicing variant allele (NM_00025.3: c.732+G>A) was identified in this fetus and her mother, not her father, indicating a maternal inheritance. Subsequently, direct sequencing confirmed the presence of this splicing variant among her grandmother (mother of mother), mother, older sister, and herself in a heterozygous manner. No PCR products were amplified by qRT-PCR for the RNA samples extracted from peripheral blood cells. In addition to this proband who was diagnosed with EA, her older sister and grandmother (mother of mother) were diagnosed with isolated asymptomatic LVCN, but her mother was just a carrier with no marked clinical manifestations after family screening.

**Conclusion:**

The presence of *MYH7* splicing variant c.732+G>A can be inherited maternally, and its cardiac phenotypes are different from one carrier to another.

## Background

Left ventricular non-compaction cardiomyopathy (LVNC) is an increasingly recognized type of cardiomyopathy that is characterized by excessive trabeculations of the left ventricle (LV) [1]. Recent studies have shown that the clinical symptoms of LVNC range from severe prenatal manifestations to asymptomatic cardiomyopathy [2]. Although the mechanism of LVNC is not completely known, most researchers assume that genetics play a conspicuous part in the long-term outcomes of patients with LVNC and their families. Furthermore, LV systolic dysfunction is connected to genetics [3]. Some patients with Ebstein's anomaly (EA) associated with LVNC have mutations in *MYH7*, which is the most frequently mutated gene in EA [4]. Consequently, due to the variable penetrance of autosomal dominant inheritance [5], genetic testing and prompt screening are not limited to at-risk relatives.

We describe a fetus with LVNC combined with EA, perimembranous ventricle septal defects (VSD), mild seroperitoneum and single umbilical artery (SUA). A *MYH7*-specific splice site mutation that segregates with the cardiac abnormalities was observed in the at-risk first-degree relatives of the fetus.

## Methods

### Fetal ultrasound and echocardiography examination

The ultrasound examinations were performed using the General Electric Voluson E8 or E10 ultrasound system with transabdominal 2.0–5.0 MHz curvilinear transducers (GE Healthcare Ultrasound, Milwaukee, WI, USA). A complete fetal echocardiographic examination, including two-dimensional (2D), M-mode, color, and pulse Doppler echocardiography, was performed according to the ISUOG Practice Guidelines and standards for performance of the fetal echocardiogram [6].

### Whole-exome sequencing (WES)

WES was performed as previously described [7–9]. Briefly, gDNA was extracted, hybridized, and enriched for sequencing. Then, we sequenced the captured libraries using Illumina NovaSeq 6000. Next, we used BWA (http://bio-bwa.sourceforge.net/) to align the sequencing data to the human reference genome (hg19/GRCh37) and Picard (http://picard.sourceforge.net/) to remove PCR duplicates. We applied GATK (https://software.broadinstitute.org/gatk/) to call variants and ANNOVAR (http://wannovar.wglab.org/) to annotate and interpret variants. Variants were filtered out if their frequencies were greater than 0.05% in the GnomAD database. We then evaluated each variant considering a careful review of the literature and in silico prediction tools (SIFT, Polyphen2, and Mutation Taster for missense variants and MaxEntScan, GeneSplicer, and Human Splicing Finder for splicing variants). We determined the pathogenicity of variants according to the American College of Medical Genetics and Genomics guidelines [10].

## Results

### Clinical phenotypes

A 33-year-old gravida 3 para 1 pregnant woman who was diagnosed with fetal anomaly at their local hospital was referred to our hospital at 24^+2^ weeks gestation.

**Fig. 1:**
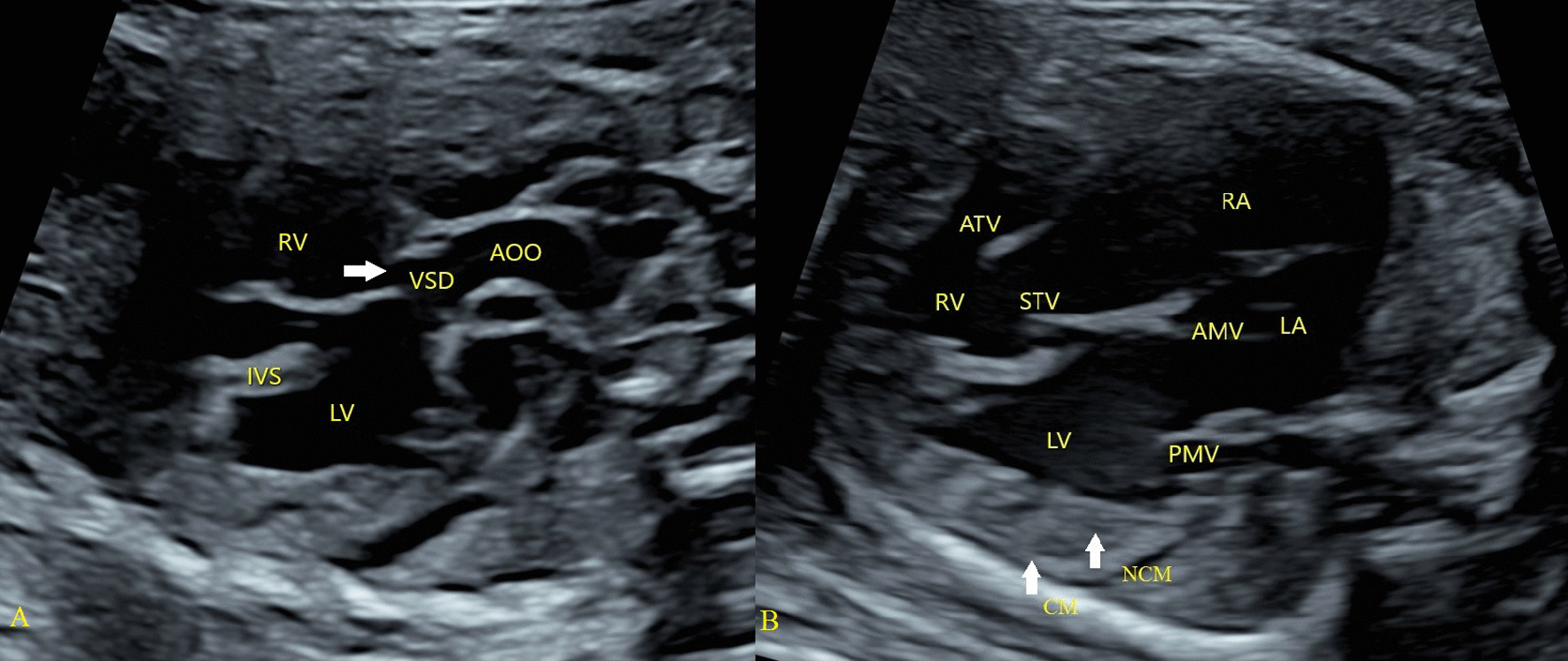
Phenotype of the fetus: perimembranous ventricle septal defect (**A**); Ebstein's anomaly, left ventricular noncompaction (**B**). **A** The ventricle septal defect (arrow) was located below the aortic valve. **B** EA (The displacement of the septal tricuspid leaflet from the mitral valve annulus was greater than 0.3 cm); left ventricular noncompaction (arrow) and heart dilatation. *VSD* ventricle septal defect, *AAO* ascending aorta, *LV* left ventricle, *LA* left atrium, *RV* right ventricle, *RA* right atrium, *CM* compact myocardium, *NCM* non-compact myocardium, *IVS* interventricular septum, *STV* septum tricuspid valve, *ATV* anterior tricuspid valve, *AMV* anterior mitral valve, *PMV* posterior mitral valve

The mother of the fetus was normal and did not take any medication during her pregnancy. Second trimester ultrasound and echocardiography showed several malformations: EA (the displacement of the septal tricuspid leaflet from the mitral valve annulus was 0.58 cm) with severe tricuspid regurgitation, heart dilatation, perimembranous VSD (Fig. 1). The fractional shortening (FS) was reduced to 11.3%. Mild seroperitoneum, SUA, and slight pericardial effusion were observed. After detailed counseling, the couple decided to terminate the pregnancy based on the genetic tests and have an autopsy. On gross pathological examination, the myocardial wall of the LV was thick and loose, accompanied with elongated anterior tricuspid leaflet, mild displacement of posterior and septal tricuspid. Histologically, there was focal necrosis of the heart muscle and pathological pigmentation.

The older sister of the fetus had healthy growth and development and had never received any cardiac evaluation ever before. The maternal grandmother of the fetus and family members had no symptoms of arrhythmias or major events, such as heart failure, thromboembolism, or sudden cardiac death.

**Fig. 2:**
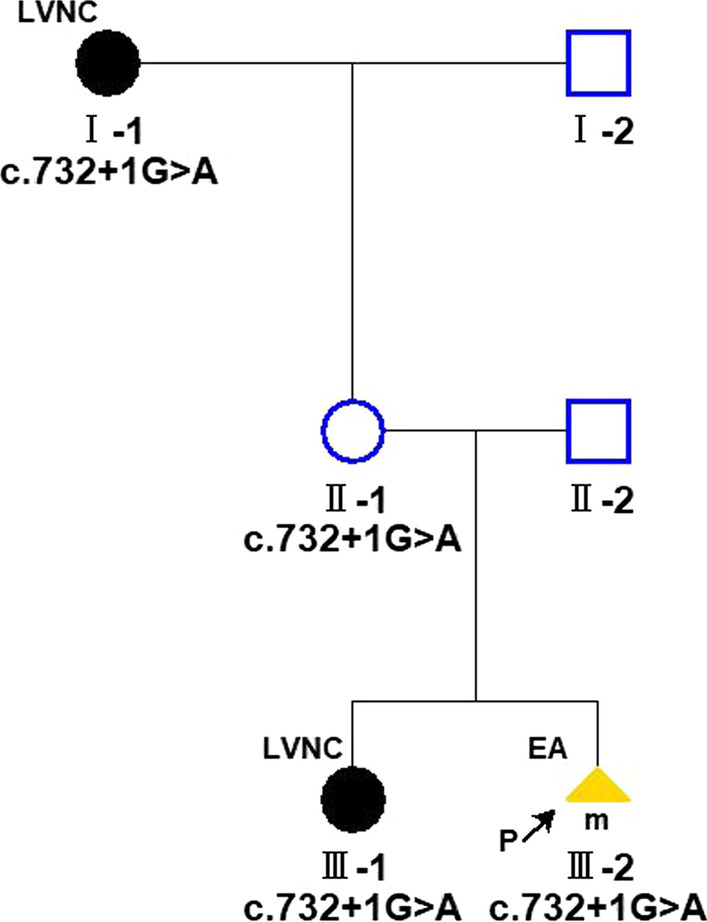
Black round symbols indicate individuals with asymptomatic LVNC; Yellow triangle symbols, individual with the presence of EA

**Fig. 3:**
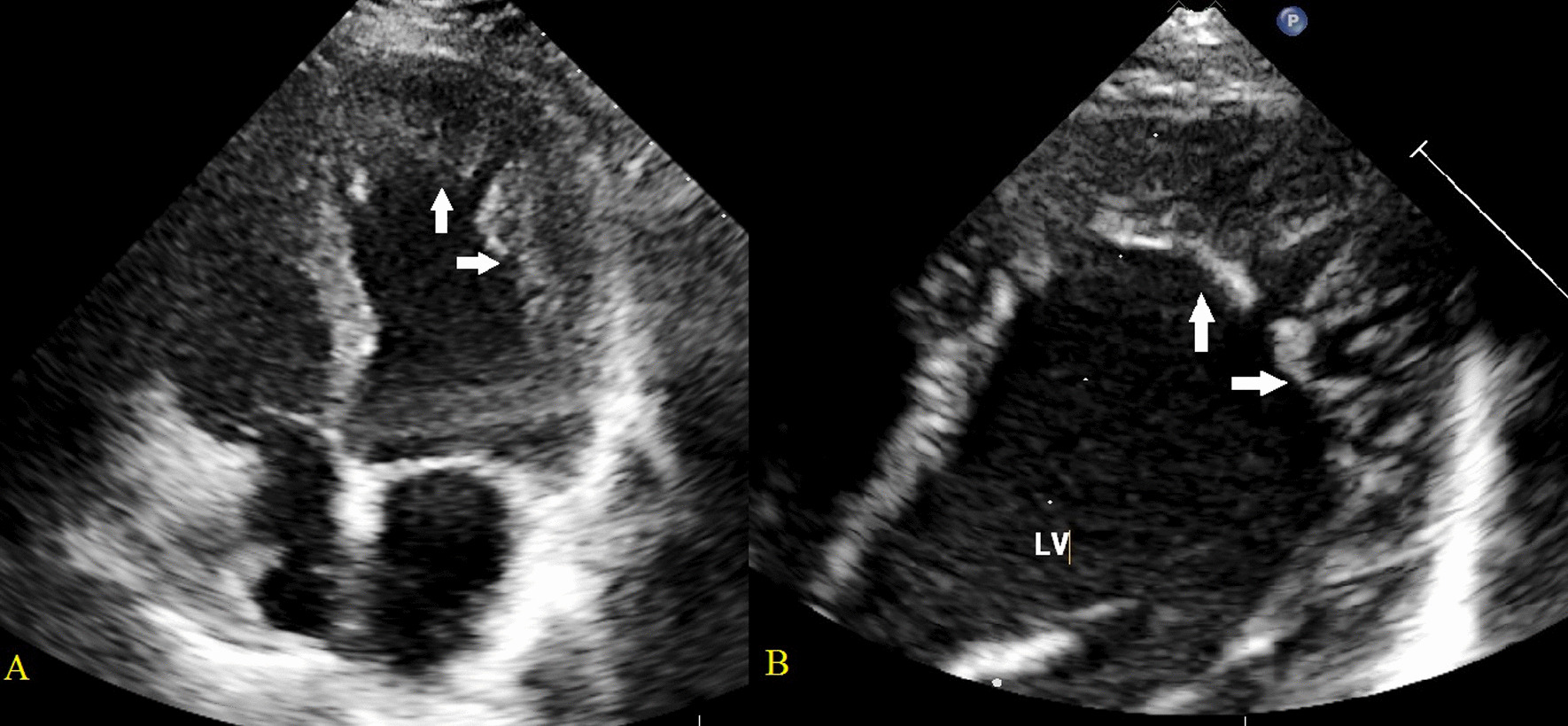
Transthoracic echocardiographic images. Apical 4-chamber views demonstrating LVNC with excessive trabeculations (arrow) in the grandmother (**A**) and older sister of the fetus (**B**)

Transthoracic echocardiography was performed to assess LV size, LV wall thickness, systolic and diastolic function, and to look for any associated CHD. Echocardiography performed on the pregnant woman indicated normal cardiac anatomy, but we found the older sister (III-1) and grandmother (mother of mother) (II-1) of the fetus were LVNC, which was characterised by left conspicuous ventricular trabeculations and sinusoids communicating with its cavity on the apical four-chamber view, particularly in their apex (Fig. 2). The end-systolic ratio of noncompacted to compacted (NC/C) myocardium was > 2.0 at the end of diastole, which met the Jenni echocardiography criteria [11] (Fig. 3). Mild mitral and tricuspid regurgitation was also observed in both of these individuals (III-1 and II-1). The LV size was normal, and the preserved systolic and diastolic functions were preserved. No intracardiac thrombi were found. The electrocardiogram of the maternal grandmother (II-1) was abnormal and showed a T-wave inversion. Considering that the LVNC was isolated and asymptomatic, cardiovascular magnetic resonance (CMR) imaging was not performed for the older sister (III-1) or maternal grandmother (II-1) of the fetus.

### Molecular findings

**Fig. 4:**
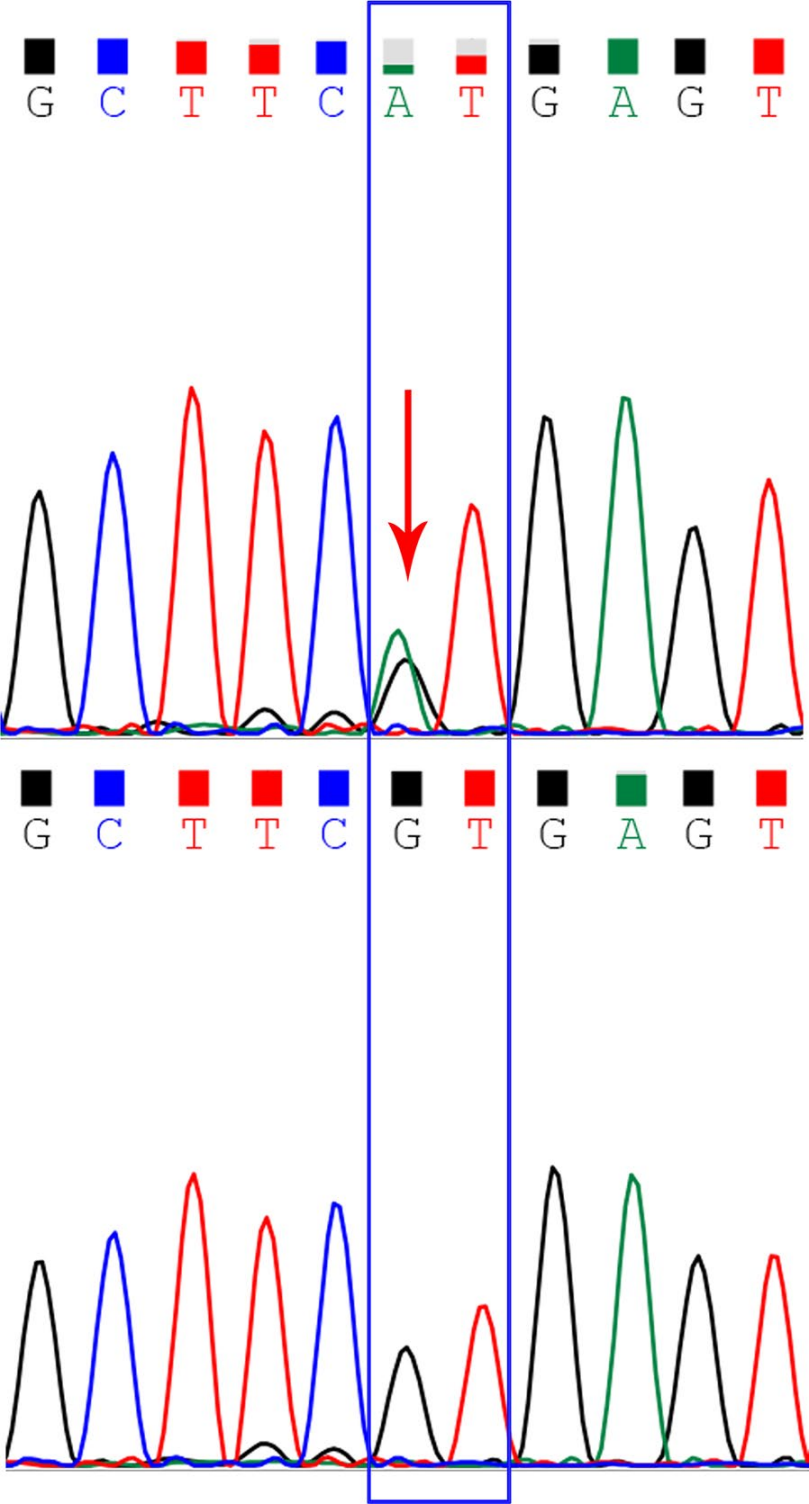
Sanger sequencing confirmed that the pregnant woman carried the MYH7 gene mutation (upper panel, red arrow), while her husband did not (lower panel). The mutation replaced the canonical splice donor sequence GT to GA (blue box), which is expected to disrupt RNA splicing and likely result in an absent or disrupted protein product. Primers used in this study were as below: chr14-23900793-F: ATGGCACTCACAGGTCTCTATG; chr14-23900793-R: GTACTTTGCTGTTATTGCAGCC. The length of the PCR product was 415 bp

A trio (fetus and the parents) WES identified a maternal inherited heterozygous splice site mutation in *MYH7* (NM_000257.3:c.732+1G>A). This splicing mutation affects the donor splice site in intron7 (+ 1 splice donor of exon 8) of the *MYH7* gene. It has been reported previously in several individuals with LVNC and one individual with isolated EA [12–15]. In contrast, it is present in only one individual (allele frequency: 3.98e-6) in the gnomAD database (https://gnomad.broadinstitute.org). This variant is reported as pathogenic in ClinVar, and it shows a deleterious effect by multiple in silico algorithms. In summary, the variant is classified as likely pathogenic according to the American College of Medical Genetics and Genomics guidelines [10]. Subsequent Sanger sequencing confirmed that the mutation was heterozygous in the fetus, her maternal grandmother, her mother and her older sister (Fig. 4). The *MYH7* mutation segregated with cardiac abnormality in the family and was observed in 3/3 affected individuals, where a blood sample was available, and in one apparently healthy individual.

In addition to finding the *MHY7* mutation presented above, we also analyzed variants in genes associated with CHD or cardiomyopathy. No pathogenic/likely pathogenic variants in these genes were identified.

## Discussion

The incidence of LVNC in all echocardiographic patients is approximately 1–3%, and approximately 20–40% of cases tend to be familial and hereditary [16, 17]. Currently, echocardiography is the main diagnostic method for LVNC. The prenatal ultrasound examination showed cardiomegaly, with features indicating noncompaction of the myocardium apparent in the third trimester [13, 18, 19].

When we reviewed the echocardiography for the fetus, we evaluated the tricuspid valve leaflets, the function of both ventricles, and the diagnosis of hydrops combined with cardiomegaly, which was caused by the severe tricuspid valve insufficiency of EA. However, the hypertrophic dilated LVNC diagnosis was missed. This was a mixed phenotype characterized by left ventricular thickening and dilation at presentation. We attributed EA to abnormal dilation and systolic and diastolic function but did not consider myocardial problems. This suggests that in the process of echocardiography, in addition to paying attention to the examination of structural deformities, we should also focus on the morphological and functional changes of the myocardium.

Despite the progress in the diagnosis and treatment of this disorder in recent years, the genetic causes are still being explored. Several genes encoding sarcomeres and cytoskeletal components, such as *TTN* and *MYH7*, have been identified to cause LVNC [20]. Mutations in the sarcomere-encoding gene *MYH7* were associated with LVNC and EA, and the frequency of *MYH7* mutations was significantly different between probands [5].

This familial case emphasizes the striking cardiac phenotypic variability associated with the c.732+1G>A spice site variant. Our index case presented as an EA. However, her older sister and maternal grandmother showed an isolated asymptomatic LVNC during the familiar screening, and her mother had no clinical manifestations. The mechanism underlying the variable penetrance in dominant gene mutations, such as the *MYH7* mutation reported here, is not clear. The influence of the internal and external environment of organisms on gene expression may be an important factor of penetrance, similar to individual genetic background. Our report also emphasizes the importance of genetic and clinical screening of the relatives of the proband.

*MYH7* splice site variants have generally been considered nonpathogenic and are not associated with cardiomyopathy [21]. However, the c.732+1G>A splice donor variant has previously been reported to occur in multiple unrelated patients with LVNC and one patient with EA [21]. These findings indicate that c.732 + 1 is a mutational hot spot that has mutated recurrently in LVNC [15]. It was significantly enriched over the general population rate in gnomAD (1/251490). These data suggest that this mutation, although located at the splice site, is associated with LVNC/EA. This is unlike other nonpathogenic *MYH7* splice site mutations [22].

The *MYH7* c.732+1G>A mutation variant is located in the region of the splice consensus sequence. It is predicted to cause altered splicing, leading to an abnormal or absent protein. Loss-of-function mutations, such as the c.732+1G>A mutation reported here, have previously been considered nonpathogenic and are not associated with cardiomyopathy [23]. In contrast, most pathogenic mutations in *MYH7* are missense variants [24, 25], indicating that haploinsufficiency may not be sufficient to cause disease. We speculate that this mutation is unique and may cause LVNC through a currently unclear mechanism. Additional evidence is required to definitively determine its clinical significance and the exact mechanism of action of this mutation.

In terms of phenotypes, the *MYH7* c.732+1G>A mutation has been reported in several individuals with LVNC, and it segregated with disease in all the affected relatives from 3 families [11, 12, 20, 26], These findings suggest that the mutation appears to have complete penetrance. However, the mother of the proband was a carrier of that splicing variant examined. We cannot exclude the possibility that she develops adult-onset cardiomyopathy later in life. This could also be caused by the nonpenetrance of the mutation. In addition, the phenotypes in the family reported here also varied greatly, with the proband being prenatally diagnosed with EA, two carriers having asymptomatic LVNC (older sister and maternal grandmother of the proband) and one unphenotypic carrier. This report demonstrates the complexity of the genotype–phenotype association in genetic cardiomyopathy. We identified more asymptomatic patients by genetic screening for family members of this fetus, which facilitates early intervention and demonstrates the importance of family screening for genetic cardiomyopathy [27].

## Conclusion

Our report suggests that the presence of *MYH7* splicing variant c.732+G>A can be inherited maternally, and that its cardiac phenotypes may vary among individual carriers.

## Data Availability

The data that support the findings of our study are included in our article and additional supporting files. The raw data of whole-exome sequencing of the family in this study are not publicly available in order to protect participant confidentiality, but are available from the corresponding author on reasonable request. If you want to request access to the data, please contact Prof. Yihua He (Email: heyihuaecho@hotmail.com) at Department of Echocardiography and Maternal–Fetal Consultation Center of Congenital Heart Disease in Beijing Anzhen Hospital, Capital Medical University, Beijing, China, or contact Dr Suzhen Ran (Email: ransuzhen0000@163.com) at Department of Ultrasound, Chongqing Health Center for Women and Children, Chongqing, China.

## Abbreviations

LVNC: Left ventricular non-compaction cardiomyopathy
CHD: Congenital heart defect
WES: Whole-exome sequencing
EA: Ebstein's anomaly
VSD: Ventricle septal defects
SUA: Single umbilical artery
LV: Left ventricle
FS: Fractional shortening
RV: Right ventricle
CMR: Cardiovascular magnetic resonance
